# Inhibition of Cyclin D1 by Novel Biguanide Derivative YB-004 Increases the Sensitivity of Bladder Cancer to Olaparib via Causing G0 / G1 Arrest

**DOI:** 10.7150/ijbs.105072

**Published:** 2025-02-18

**Authors:** Di Xiao, Xuetong Chu, Weifan Wang, Mei Peng, Qi Lv, Cangcang Xu, Huaxin Duan, Xiaoping Yang

**Affiliations:** 1 Key Laboratory of Study and Discovery of Small Targeted Molecules of Hunan Province, Department of Oncology, Hunan Provincial People's Hospital, The First Affiliated Hospital of Hunan Normal University, The Research Center of Reproduction and Translational Medicine of Hunan Province, Key Laboratory of Chemical Biology & Traditional Chinese Medicine Research of Ministry of Education, School of Pharmaceutical Sciences, Health Science Center, Hunan Normal University, Changsha 410013, Hunan, China.; 2 FuRong Laboratory, Changsha 410078, Hunan, China.; 3 TCM and Ethnomedicine Innovation and Development International Laboratory, Innovative Material Medical Research Institute, School of Pharmacy, Hunan University of Chinese Medicine, Changsha, China.

**Keywords:** Biguanides, Cell cycle, Homologous recombination, PARP inhibitor

## Abstract

Bladder cancer (BC) is the 10^th^ most common type of tumor worldwide, and recently approved immunotherapies and FGFR inhibitors have been shown to improve the prognosis of only a very limited subset of BC patients. Thus, the quest for more effective drugs remains an urgent priority for improving the quality of life of more BC patients. Previously, we demonstrated that a novel biguanide **YB-004** has potent antitumor activity. In this study, we found that the novel biguanide **YB-004** interrupts the cell cycle by reducing the expression of cyclin D1, causing G0/G1 phase arrest, and suppresses homologous recombination (HR) by inhibiting Rad51, thereby increasing DNA damage and blocking BC cell proliferation. Interestingly, our results further revealed that cell accumulation in the S and G2/M phases is the main reason why HR-proficient BC cells are not sensitive to olaparib, as these phases are conducive to HR activation and DNA repair. Thus, **YB-004** increased the sensitivity of BC cells to olaparib by reversing the cell cycle changes and HR activation caused by olaparib. Taken together, these findings suggest that the combination of **YB-004** with olaparib has great potential for the clinical treatment of HR-proficient BC.

## Background

According to global cancer statistics, bladder cancer (BC) is the fourth most common cancer in men, with a mortality rate ranking in the top ten 1, 2. In recent years, targeted drugs such as the FGFR3 inhibitor erdafitinib and immunotherapies such as the PD-1/PD-L1 inhibitors nivolumab and atezolizumab have been approved for the treatment of BC, bringing new hope to BC patients 3, 4. However, subsequent clinical data have shown limitations such as unclear improvements in efficacy, low response rates and the development of resistance 5, 6. Therefore, there is an urgent need to explore new, highly effective and low-toxicity targeted therapeutic strategies for BC.

Owing to their superior antitumor effects, biguanides have attracted widespread attention both domestically and internationally 7-9. The "miracle drug" metformin has shown significant antitumor activity in various cancer types, such as pancreatic cancer and prostate cancer 10-12. To date, despite over 400 clinical trials investigating the antitumor effects of metformin, its clinical application for cancer treatment has not yet been approved. One of the main reasons is that high concentrations of metformin are needed to kill cancer cells, making it difficult to translate this drug into clinical treatments 13. Therefore, further measures are needed to increase the antitumor activity of biguanide drugs. In our previous study, we successfully used intermediate derivatization methods 14 to optimize the structure of biguanides, obtaining a series of novel derivatives, among which **YB-004** has shown superior biological activity in BC cells and has high potential for clinical application 15. Therefore, further in-depth research into the antitumor mechanisms of **YB-004** is necessary.

The activation of AMPK to regulate mitochondrial energy metabolism is the most common mechanism by which biguanide drugs exert beneficial clinical effects 16, 17. However, some new studies suggest that the AMPK signaling pathway is not the only pathway through which biguanide drugs exert their antitumor effects. For example, Sahra *et al.* demonstrated that metformin inhibits cell proliferation by downregulating Cyclin D1, leading to cell cycle arrest at the G0/G1 phase and preventing cell proliferation 18. Cyclin D1 plays a pivotal role in cell cycle regulation as a key protein that governs the progression of the cell cycle 19, 20^.^ By partnering with CDK4/6, cyclin D1 aids in the transition from the G1 phase to the S phase through Rb protein phosphorylation. This action liberates E2F transcription factors, fostering cell cycle progression. Moreover, cyclin D1 influences cell proliferation and growth by modulating the expression levels and activity of critical cell cycle regulators 21, 22. In addition to its involvement in managing the cell cycle and proliferation, cyclin D1 is associated with the activation of the DNA damage response and repair signaling pathways. For example, Jirawatnotai S *et al.* reported that inhibiting the expression or function of Cyclin D1 increases radiation-induced DNA damage and triggers cell death by blocking HR 23. Blocking the activation of HR is also one of the main pathways that sensitizes HR-proficient patients to DNA damage drugs such as PARP inhibitors. Therefore, recent research has focused on combination therapy strategies using drugs that inhibit HR activation to expand the use of PARP inhibitors to a larger population of HR-proficient patients. However, to date, there are no direct inhibitors that specifically target the proteins that catalyze HR 24. Thus, increasing the sensitivity of PARP inhibitors by indirectly inhibiting HR through the downregulation of Cyclin D1 is a promising strategy. However, the specific mechanism by which Cyclin D1 regulates HR is not yet fully understood. In the present study, we showed that cyclin D1 facilitated HR activation during G2/M arrest by increasing the expression of Rad51, a pivotal protein in the HR pathway. Notably, **YB-004** suppressed cyclin D1 expression, inducing G0/G1 arrest and reducing Rad51 expression to inhibit HR. Therefore, the synergistic effect of **YB-004** with olaparib, a prominent PARP inhibitor, results in "synthetic lethality" and proves to be a potent strategy for the treatment of HR-proficient BC.

## Materials and methods

### Cell culture

T24, RT4, HUVEC were obtained from iCell Bioscience Inc (Shanghai, China) and validated for authentication using the short tandem repeat (STR) method. T24 and RT4 cells were cultured in 5A with 10% fetal bovine serum (FBS). HUVEC cells were cultured in MEM with 10% FBS. All cells were incubated at 37 °C in a 5% CO_2_ atmosphere.

### Mice model

BALB/C-nude mice (female, 4-6 weeks of age, weighting 18-20 g) were provided from Hunan Silaikejingda Experimental Animal Co., Ltd. (Changsha, Hunan, China). BALB/C-nude mice were used to detect the *in vivo* antitumor activity of **YB-004**, Olaparib, and their combination on the growth of T24 cells. For *in vivo* antitumor assay, T24 cells were injected into the right flank of each BALB/C-nude mouse.

The tumor-bearing mice were randomly divided into four groups (n=5 /group). Olaparib (50 mg/kg), **YB-004** (6 mg/kg), and the combination of **YB-004** (6 mg/kg) and Olaparib (50 mg/kg) were administered by intraperitoneal injection for 14 consecutive days. Tumor volume was recorded every day. After 14 days of treatment, the mice were killed to detect the weight of tumors in each group. Tumor volume was measured with a Vernier caliper.

### Cell viability assay

Cells were seeded into 96-well plates at a density of 5×10^3^ cells /well, then treated or not with drugs (Olaparib, **YB-004** or the combination of the both) for 72 h at increasing concentrations. At the end of time point, cells were incubated with 0.5% MTT for 4 h at 37℃. The supernatant was then discarded, the MTT was dissolved with 150 μL of DMSO and absorbance read at OD = 550 nm.

### Western blot analysis

Equal amounts of total proteins were loaded for SDSPAGE and transferred onto a PVDF membrane. Membranes with protein were blocked with 5% (w/v) skim milk, incubated with primary antibody in Supplementary Materials, and then incubated with secondary antibodies (1: 2000) for detection. β-Actin were used to normalize the level of protein expression. Grayscale was measured by Image J (National Institutes of Health, https://imagej.net/ij/features.html).

### RNA extraction and quantitative real-time-PCR (qRT-PCR)

RNA was isolated for qRT-PCR was performed using Trizol reagent. Complementary DNA (cDNA) was synthesized using a high-capacity cDNA reverse transcription kit (Vazyme, Nanjing, China). qRT-PCR was carried out using a TaqMan Gene Expression Master Mix (Vazyme, Nanjing, China) according to manufacture protocol. The sequences of primers are shown in Table S1.

### Immunofluorescence assay

For the silencing groups, RT4 and T24 cells stably expressing Cyclin D1 (CCND1) shRNA and NC shRNA were treated with Olaparib for 12 h, 24 h and 48 h. For the drug-treated group, RT4 and T24 cells were treated with **YB-004**, Olaparib or their combination for 12 h, 24 h and 48 h. After treatment, cells were fixed with 4% formaldehyde, permeabilized with 0.2% (v/v) Triton X-100 in PBS, blocked with 1% (w/v) bovine serum albumin (BSA) in PBS for 1.0 h, and stained with anti-p-Histone 2AX (γH2AX) antibody and anti-Rad51 (Rad51) antibody labeled with Cy3.

After staining, cellular DNA was counterstained with 4,6-diamidino-2-phenylindole (DAPI). Fluorescence signals were detected using a laser confocal microscope.

### Comet assay

In brief, for the drug-treated groups, RT4 cells and T24 cells were treated with **YB-004**, Olaparib, or their combination for 12 h, 24 h and 48 h; harvested; and combined with 0.7% low melting point agarose at a ratio of 1: 10 (v/v). Slides were immersed in a lysis solution for 30 min and electrophoresed in a horizontal electrophoresis apparatus. Five images were randomly captured per slide. The length of cells was measured by Image J (National Institutes of Health, https://imagej.net/ij/features.html).

### H&E staining

The liver and kidney tissue samples of xenograft model mice were fixed with 4% paraformaldehyde, dehydrated with ethanol, immersed in xylene, embedded in paraffin, and cut into 4.0 μm longitudinal sections. The paraffin-embedded sections were stained with hematoxylin and eosin (H&E) according to the manufacturer's instructions. Each group of samples was observed with a fluorescence microscope. Five images were randomly captured per slide.

### Immunohistochemical staining

The tumors from xenograft-model mice were embedded in paraffin and cut into longitudinal sections. The paraffin-embedded sections were incubated with 0.3% hydrogen peroxide for 30 min to block endogenous peroxidase and then incubated with 1.0% bovine serum albumin (BSA) for blocking. After blocking, the paraffin-embedded sections were incubated with the primary antibody overnight at 4 °C, incubated with secondary antibody for another 1 h at room temperature and then counterstained for 1 min with hematoxylin. Each group was examined using a fluorescence microscope. The level of protein expression was measured by Image J (National Institutes of Health, https://imagej.net/ij/features.html).

### Cell cycle

The cell cycle was analyzed by a Cell cycle assay kit (red fluorescence) (Elabsicence, Wuhan, Hubei, China). For the drug-treated groups, RT4 cells and T24 cells were treated with different concentrations of **YB-004**, Olaparib, or their combination for 24 h. After treatment, Cellular DNA was stained with PI following the manufacturer's protocol. The cell cycle was detected by BD FACSCelesta flow cytometry, the cell cycle data were analyzed by ModFit LT (Verity Software House, https://www.vsh.com).

### HR assay

The HR reporter plasmid pDRGFP and endonuclease encoding pCBASce1 (I-Sce1) (Addgene plasmids #26475 and #26477, respectively) were used. RT4 cells grown on 6-well plates were transfected with the plasmids using Lipofectamine 6000 according to manufacturer's instructions, and then treated with vehicle, **YB-004**, Olaparib or **YB-004** combined with Olaparib for 24 h as described above and GFP-positive cells were measured by BD FACSCelesta flow cytometry. GFP positive rate was measured by Flow Jo (BD, https://www.flowjo.com/).

### shRNA lentivirus infection

Human recombinant CCND1 shRNA lentivirus and the negative-control (NC) shRNA lentivirus were constructed by GeneChem Co., Ltd. (Shanghai, China). RT4 and T24 cells were infected with CCND1 shRNA1, CCND1 shRNA2, and NC shRNA lentiviruses using HitransGP promoting reagent according to the manufacturer's instructions. Three days after RT4 and T24 cells infection with shRNA lentiviruses, the expression of CCND1 was measured by Western blot. Puromycin was used to select for RT4 and T24 cells stably expressing CCND1 shRNA and NC shRNA. The sequences are listed in Table S2.

### Quantification and statistical analysis

The data were analyzed using Graphpad prism 8. The results are expressed as means ± SD. Differences between treatment regimens were analyzed by two-tailed Student's t-test or one-way ANOVA. *P < 0.05; **P < 0.01; ***P < 0.001.

## Results

### YB-004 inhibited the expression of Cyclin D1 and blocked the proliferation of BC cells

**Figure 1:**
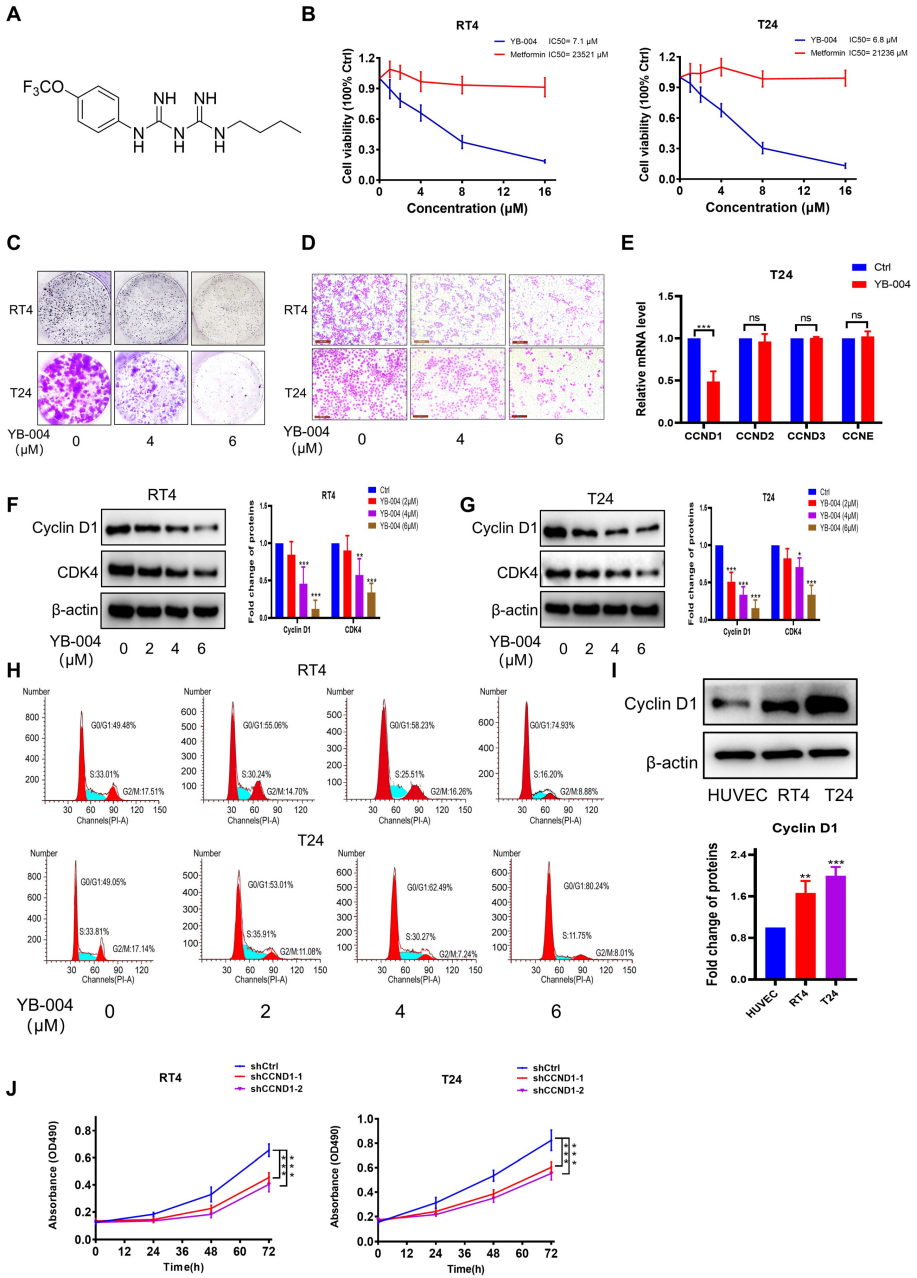
** YB-004 induced G0/G1 arrest to inhibit proliferation of BC cells. (A)** Chemical structure of** YB-004**. **(B)** The cell viability after** YB-004** or metformin treatment were evaluated by MTT assay. **(C-D)** The inhibitory effects of **YB-004** on proliferation and migration of RT4 and T24 cell lines were detected by colony formation and transwell assay. **(E-G)** Cells were treated with **YB-004** for 24h, and the changes of the indicated mRNA or proteins were analyzed by RT-PCR or WB. **(H)** Cells were treated with **YB-004** for 24h, the changes of cells cycle were detected by flow cytometer. **(I)** The protein expression of Cyclin D1 in different types of BC cell lines and HUVEC. **(J)** The proliferation of shCtrl cells and shCCND1 cells were detected by MTT assay (n=3, Error bars represent means ± SD from triplicate experiments, *P < 0.05; **P < 0.01; ***P < 0.001).

Our laboratory previously reported that metformin significantly inhibits the proliferation of BC cells 13, 17, 25. However, the concentration of metformin required for antitumor effects is high, making it difficult to achieve the necessary blood drug levels through oral administration 26. To address this challenge, our laboratory proposed the intravesical instillation of metformin to increase the local concentration at the tumor site 17. Additionally, by using intermediate derivatization methods 15, 27, we synthesized a series of novel biguanide derivatives in an attempt to obtain increased antitumor activity through structural modifications. Fortunately, we found that **YB-004** (**Figure 1A**) exhibited potent anti-proliferative activity in RT4 and T24 BC cells, with approximately 3000 times greater activity than that of metformin (**Figure 1B**). Furthermore, **YB-004** significantly inhibited the colony formation and migration of BC cells (**Figure 1C-D**). Overall, these results demonstrate the significant potential of **YB-004** in treating BC.

Given the promising antitumor effects of **YB-004** in BC cells, we further investigated the antitumor mechanism of **YB-004**. As mentioned earlier, cell cycle inhibition is one of the important mechanisms of the antitumor effects of metformin, and CCND plays a crucial role in cell cycle regulation during the middle phase. Therefore, we first conducted PCR to investigate which subtype of CCND is affected by the new biguanide **YB-004**. Interestingly, our results revealed that **YB-004** significantly downregulates the expression of CCND1 (**Figure 1E**). Western blot results also demonstrated that **YB-004** effectively reduced the expression of Cyclin D1 in RT4 and T24 cells (**Figure 1F-G**). Cell cycle analysis revealed that cells treated with **YB-004** were arrested in the G0/G1 phase, suggesting that **YB-004** interferes with the cell cycle progression of RT4 and T24 cells (**Figure 1H**). Additionally, we analyzed the protein expression of Cyclin D1 in bladder cancer cells. The results revealed high expression of Cyclin D1 in bladder cancer cells (**Figure 1I**), and silencing Cyclin D1 significantly slowed cell proliferation (**Figure 1J**), indicating that Cyclin D1 is an effective potential target for treating bladder cancer. The above results indicate that **YB-004** induces G0/G1 phase arrest of the cell cycle by inhibiting Cyclin D1 expression, thereby inhibiting the proliferation of BC cells.

### YB-004 inhibits HR in HR-proficient BC cells, exacerbating DNA damage

**Figure 2:**
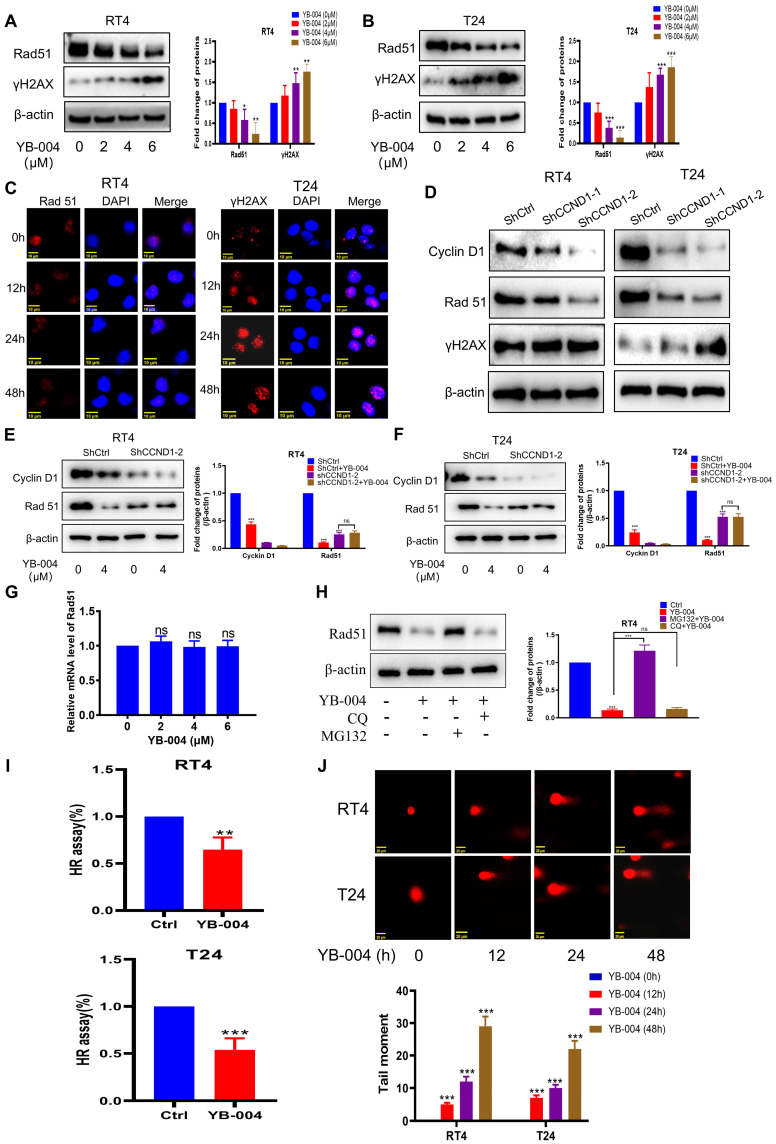
** YB-004 inhibited HR of HR-proficient BC cells exacerbating DNA damage. (A-C)** Cells were treated with **YB-004**, and the changes of the indicated proteins were analyzed by WB and immunofluorescence. **D**. Cells were transfected with lentiviral vectors and screened by puromycin. The changes of the indicated proteins were analyzed by WB. **(E-F)** Cells expressing shCtrl or shCCND1-2 treated with the **YB-004** for 24h, and the changes of the indicated proteins were analyzed by Western blot. **(G)** T24 were treated to YB-004 for 24h, and the changes of the Rad51 mRNA were analyzed by PCR. **(H)** RT4 were pretreated with chloroquine (CQ, 5µM) or MG132 (0.1µM) for 12 hours, and cells were then treated with **YB-004** for 12 hours. The changes of Rad51 were detected by WB. **(I)** cells were treated with **YB-004** for 24 h and HR was analyzed using flow cytometer. **(J)** Cells were treated with **YB-004** for 24 h and the degree of DNA damage was measured by comet assay (n=3, Error bars represent means ± SD from triplicate experiments, *P < 0.05; **P < 0.01; ***P < 0.001).

Due to the critical role of Cyclin D1 in regulating homologous recombination, we further investigated the effect of YB-004 on the expression of Rad51, which plays a key role in homologous recombination and DNA repair processes. Western blot and immunofluorescence results showed that the expression of Rad51 was significantly down-regulated by **YB-004** (**Figure 2A-C** and **Supplementary Figure S1A-C**). To further confirm whether **YB-004** inhibits Rad51 by downregulating Cyclin D1 expression, we silenced Cyclin D1 and observed the changes of Rad51 protein expression, and found that Rad51 was significantly downregulated after Cyclin D1 was silenced (**Figure 2D**). Interestingly, when Cyclin D1 was silenced, the inhibitory effect of **YB-004** on Rad51 was significantly weakened (**Figure 2E-F**), indicating that **YB-004** inhibits Rad51 by downregulating Cyclin D1 expression. To further investigate the mechanism by which **YB-004** inhibits the expression of Rad51 after inhibiting cyclin D1, we first conducted PCR to observe whether **YB-004** downregulated the expression of Rad51 mRNA. Surprisingly, we found that **YB-004** had no effect on the expression of Rad51 mRNA (**Figure 2G**). Therefore, we further analyzed whether **YB-004** regulated Rad51 through post-translational mechanisms. We discovered that in the presence of the lysosomal inhibitor chloroquine, **YB-004** was still able to downregulate the expression of Rad51. However, when a proteasome inhibitor MG132 was present, the downregulation of Rad51 by **YB-004** was reversed (**Figure 2H**), indicating that after **YB-004** downregulated cyclin D1, it promotes the proteasomal degradation of Rad51 through post-translational mechanisms. Since Rad51 is essential in the HR process, we further investigated the impact of **YB-004**-induced Rad51 downregulation on HR. After transfection with the pDRGFP and pCBASce-I plasmids and treatment of the cells with **YB-004** for 24 hours, a significant reduction in HR efficiency was observed in both RT4 and T24 cells (**Figure 2I**).

DNA double-strand breaks are a major source of cellular genotoxicity. Failure to repair DNA double-strand breaks in a timely and effective manner may lead to cell death. Through comet assay experiments, we found that prolonged exposure to **YB-004** was associated with a significant increase in comet tail length (**Figure 2J**), indicating that the cells were unable to repair damaged DNA because of the inhibition of HR by **YB-004**, which led to increased DNA damage and cell death. Additionally, the expression of the DNA damage marker protein γH2AX was significantly increased after treatment with **YB-004** (**Figure 2A-C** and **Supplementary Figure S1A-C**), further supporting this conclusion. These results suggest that **YB-004** inhibits HR in BC cells, resulting in sustained DNA damage and cell death.

### Olaparib causes S and G2/M phase arrest in HR-proficient BC cells

**Figure 3:**
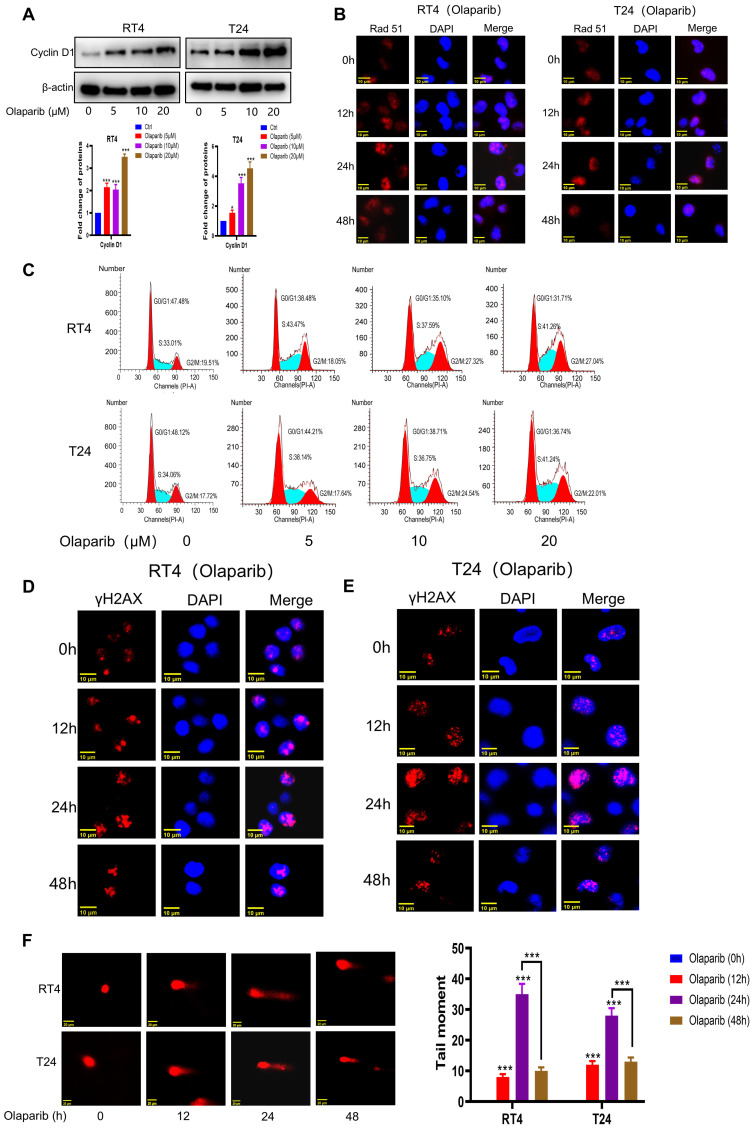
** Olaparib induced the accumulation of HR-proficient BC cells in S and G2/M phase. (A-B)** Cells were treated with Olaparib, and the changes of the indicated proteins were analyzed by WB and immunofluorescence. **C**. Cells were treated with Olaparib, the changes of cells cycle were detected by flow cytometer. **(D-E)** BC cells were treated with Olaparib and the changes of the indicated proteins were analyzed by immunofluorescence **(F)** BC cells were treated with Olaparib and the degree of DNA damage was measured by comet assay (n=3, Error bars represent means ± SD from triplicate experiments, *P < 0.05; **P < 0.01; ***P < 0.001).

**Figure 4:**
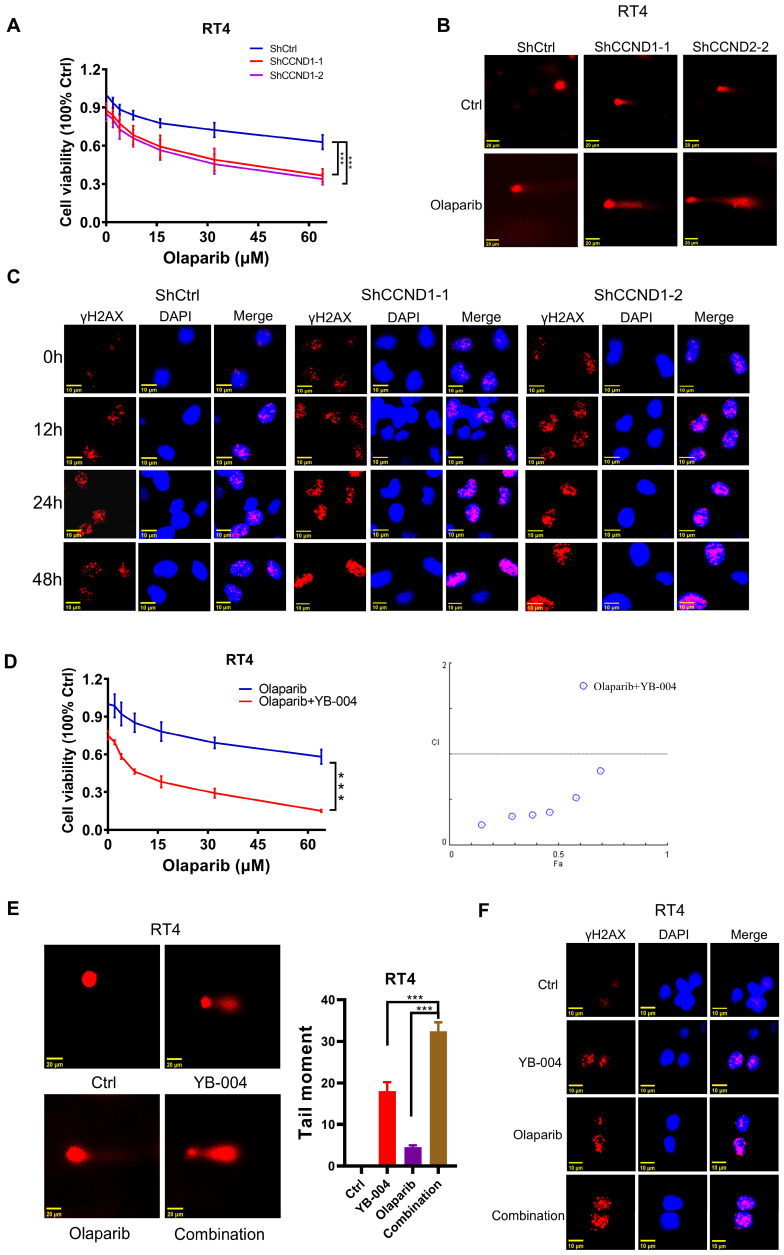
** Combination of YB-004 and Olaparib synergistically inhibits the growth of HR-proficient BC cells *in vitro*. (A)** The cell viability of RT4 expressing shCtrl or shCCND1 treated with Olaparib at different concentrations was evaluated by MTT. **(B)** RT4 expressing shCtrl or shCCND1 were treated with Olaparib for 24 h and the degree of DNA damage was measured by comet assay. **(C)** RT4 expressing shCtrl or shCCND1 were treated with Olaparib and the changes of the indicated proteins were analyzed by immunofluorescence. **(D)** The cell viability of RT4 treated with **YB-004** and Olaparib alone or in combination were evaluated by MTT. The combination index (CI) was calculated using CompuSyn software. **(E)** RT4 were treated with **YB-004** and Olaparib alone or in combination for 24 h and the degree of DNA damage was measured by comet assay. **(F)** RT4 were treated with **YB-004** and Olaparib alone or in combination and the changes of the indicated proteins were analyzed by immunofluorescence (n=3, Error bars represent means ± SD from triplicate experiments, ***P < 0.001).

HR activation is a major factor in regulating the sensitivity of genes to toxic therapies such as olaparib. It is generally believed that accurate repair by HR is limited to the S phase and the G2 phase of the cell cycle. As we previously demonstrated the important role of Cyclin D1 in regulating cell cycle progression and the HR process, we further explored whether HR-proficient BC cells that were insensitive to olaparib activated HR through the upregulation of Cyclin D1 expression. As expected, after Olaparib treatment, the expression of Cyclin D1 and the key protein involved in HR repair, Rad51, was significantly increased (**Figure 3A-B** and** Supplementary Figure S2A**). Additionally, cells accumulated in the S and G2 phases (**Figure 3C**), in which HR is more likely to occur. Consistently, our results revealed that although the expression of γH2AX was upregulated after 24 hours of olaparib treatment, the expression at 48 hours was significantly lower than that at 24 hours (**Figure 3D-E** and** Supplementary Figure S2B-C**), suggesting that while olaparib induces DNA damage in HR-proficient BC cells, the activation of HR allows the repair of damaged DNA. Our comet assay results also revealed that the intensity of DNA damage at 48 hours of olaparib treatment was significantly weaker than that at 24 hours (**Figure 3F**), indicating the need to increase the sensitivity to olaparib by inhibiting HR activation. Given the crucial role of Cyclin D1 in modulating HR, we then investigated the impact of silencing Cyclin D1 on olaparib sensitivity. The results revealed a significant increase in olaparib sensitivity after Cyclin D1 was silenced (**Figure 4A** and **Supplementary Figure S3A**). Mechanistic studies revealed that silencing Cyclin D1 led to a decrease in Rad51 expression, thereby impairing the effective repair of olaparib-induced DNA damage, which resulted in a further increase in DNA damage at 48 hours compared with 24 hours (**Figure 4B-C** and **Supplementary Figure S3B-C**). These findings suggest that inhibiting Cyclin D1 expression is an effective strategy to increase the sensitivity of HR-proficient BC cells to olaparib.

### The combination of YB-004 and olaparib synergistically inhibits the growth of HR-proficient BC cells *in vitro*

Owing to the important roles played by the upregulation of Cyclin D1 and the activation of HR in rendering BC cells insensitive to olaparib and considering our previous finding that the repression of BC cell proliferation by **YB-004** is achieved by suppressing Cyclin D1, which inhibits HR, we further investigated whether **YB-004** could also increase the sensitivity of BC cells to olaparib by inhibiting HR.

We first conducted MTT experiments to explore the synergistic anti-proliferative activity of the combination of **YB-004** and olaparib. The results revealed that combined treatment with **YB-004** and olaparib significantly inhibited the growth of RT4 and T24 cells (**Figure 4D** and **Supplementary Figure S3D**). At the same time, we also observed that **YB-004** can enhance the sensitivity of BC cells to the DNA-damaging drug cisplatin by downregulating the nuclear expression of Rad51 (**Supplementary Figure S3E-F**). These results further confirm the potential role of **YB-004** in enhancing the effects of DNA-damaging drugs such as PARPi and cisplatin by inhibiting HR. The comet assay results indicated that the comet tail length after combined treatment was significantly longer than that after single-drug treatment (**Figure 4E** and **Supplementary Figure S3G**), suggesting that the combination therapy exacerbated drug-induced DNA damage. Similarly, the immunofluorescence results demonstrated that the expression of the nuclear DNA damage marker γ-H2AX was significantly increased after **YB-004** was combined with olaparib compared to the expression after treatment with olaparib alone (**Figure 4F** and **Supplementary Figure S3H**), further indicating that the combination of **YB-004** and olaparib induced cellular DNA damage more effectively than olaparib alone. In conclusion, these results suggest that **YB-004** can increase the sensitivity of HR-proficient BC cells to olaparib, providing a potential strategy for the treatment of bladder cancer.

### Inhibition of Cyclin D1 expression by YB-004 increases the sensitivity of HR-proficient BC cells to olaparib

**Figure 5:**
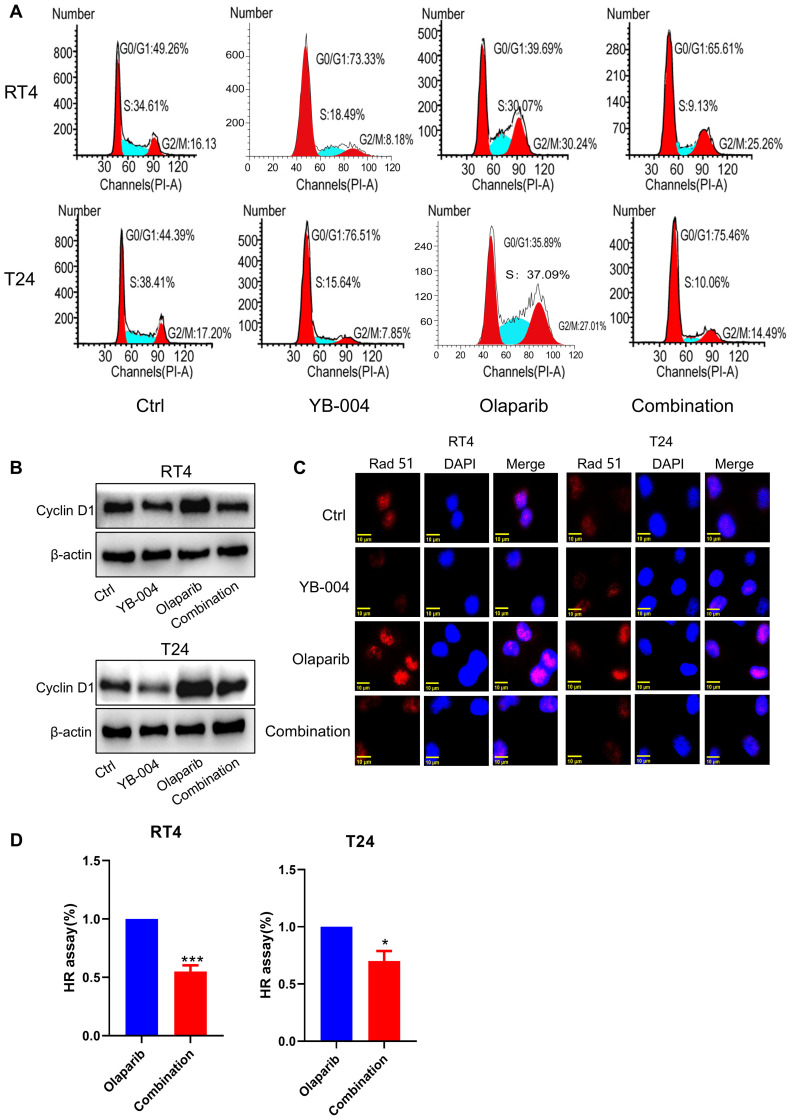
** Inhibition of Cyclin D1 expression by YB-004 enhances the sensitivity of proficient HR BC cells to olaparib. (A)** Cells were treated with** YB-004** and olaparib alone or in combination for 24 h, the changes of cells cycle were detected by flow cytometer. **(B-C)** BC cells were treated with **YB-004** and Olaparib alone or in combination and the changes of the indicated proteins were analyzed by WB or immunofluorescence. **(D)** BC cells were treated with **YB-004** and olaparib alone or in combination for 24 h and HR was analyzed using flow cytometer (n=3, Error bars represent means ± SD from triplicate experiments, *P < 0.05; ***P < 0.001).

Owing to the excellent synergistic effect of **YB-004** and olaparib in HR-proficient BC cells, we further explored the mechanism by which **YB-004** increases olaparib sensitivity. The accurate repair of HR is limited to the S phase and G2 phase of the cell cycle, so we first examined the cell cycle changes in T24 and RT4 cells. Our results revealed that **YB-004** reversed the olaparib-induced accumulation of cells in the S and G2/M phases, increasing the proportion of cells in the G0/G1 phase (**Figure 5A**). Through Western blotting, we found that **YB-004** reversed the olaparib-induced upregulation of Cyclin D1 (**Figure 5B**), leading to the downregulation of the key DNA repair protein Rad51 (**Figure 5C**). Therefore, **YB-004** can significantly inhibit the activation of HR induced by olaparib, thereby exacerbating DNA damage (**Figure 5D**). In summary, these results indicate that **YB-004** increases the sensitivity of HR-proficient BC cells to olaparib by promoting the downregulation of Cyclin D1 to inhibit Rad51 and HR.

### YB-004 and olaparib synergistically inhibited the growth of T24 xenograft tumors *in vivo*

**Figure 6:**
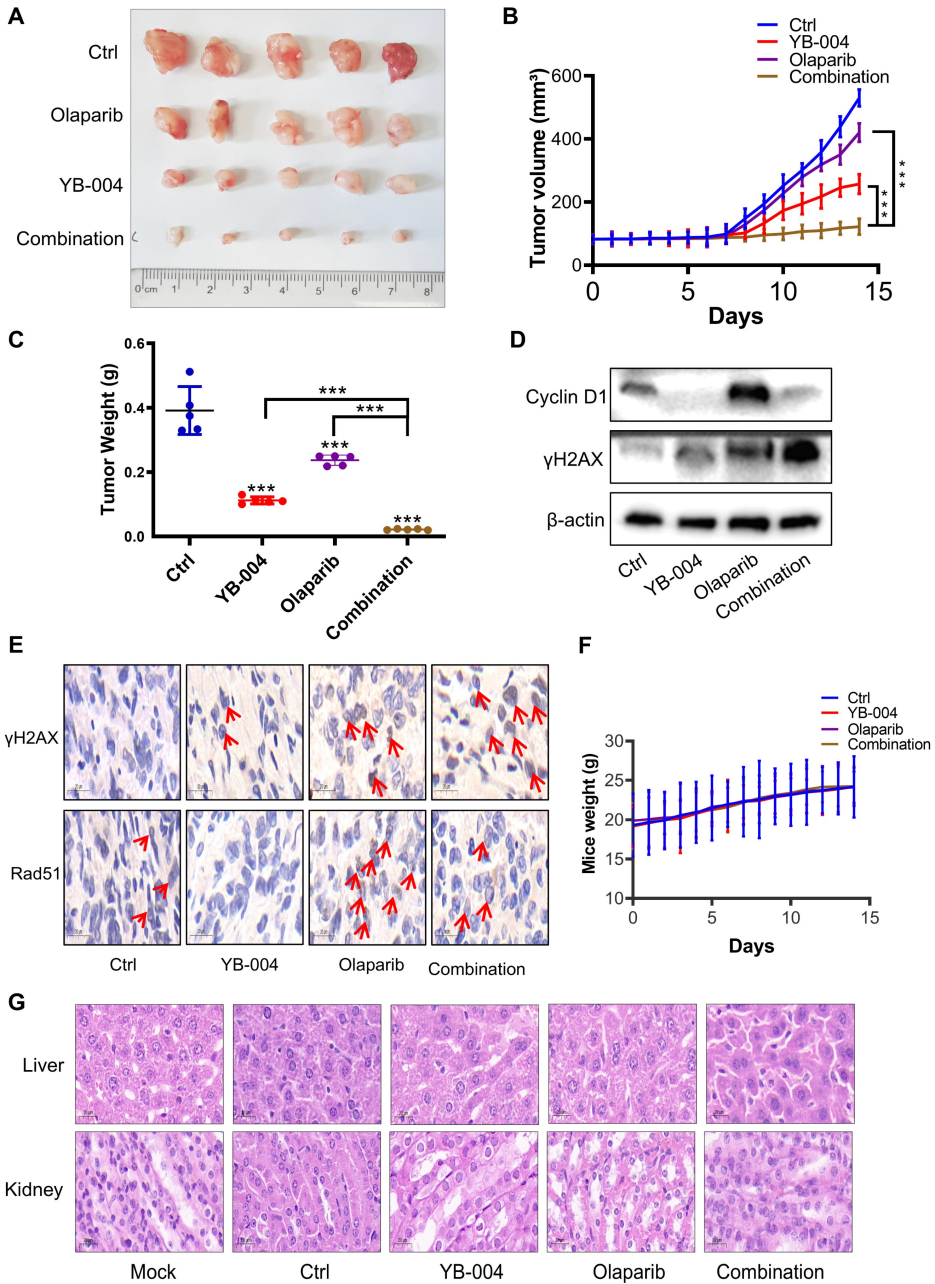
** YB-004 and Olaparib synergistically inhibited the growth of T24 xenograft tumor *in vivo*. (A-C)** T24 cells were inoculated subcutaneously into the flank of mice. When the tumor volume reached 70-100 mm^3^, mice were treated with **YB-004** and Olaparib alone or in combination. Tumor images (A), tumor volumes (B) and tumor weight (C) were then assessed (5 mice/group), ***P<0.001). **(D-E)** The expression of indicated proteins in tumor tissues after treated with **YB-004** and Olaparib alone or in combination were detected by WB and IHC. **(F)** The changes of body weight in each group of mice. **(G)** Representative images of HE analysis for liver and kidney organs of each group mice.

Considering the synergistic effect of **YB-004** and olaparib *in vitro*, we further investigated whether **YB-004** increases the sensitivity of bladder cancer cells to olaparib *in vivo*. As expected, olaparib alone had minimal inhibitory effects on tumor growth (**Figure 6A**). Interestingly, when olaparib was used in combination with **YB-004**, tumor growth was nearly halted (**Figure 6A-B**). After treatment was completed, the tumors were collected from the mice. The average tumor weight in the combination group was significantly lower than that in any single-treatment group (**Figure 6C**). These results indicate that **YB-004** significantly increases sensitivity to olaparib *in vivo*.

We subsequently explored the mechanism by which the combination of **YB-004** and olaparib inhibits the proliferation of BC cells *in vivo*. The upregulation of Cyclin D1 and Rad51 expression was also found to be a reason for the insensitivity of BC cells to olaparib *in vivo*. Interestingly, **YB-004** significantly inhibited the olaparib-induced upregulation of Cyclin D1 and Rad51 expression (**Figure 6D-E**). Importantly, although **YB-004** increased the sensitivity of BC to olaparib *in vivo*, no weight loss or liver or kidney toxicity was observed in any of the treatment groups (**Figure 6F-G**), indicating the safety of these treatments. These results further support the use of **YB-004** in combination with olaparib for the clinical treatment of HR-proficient bladder cancer patients.

## Discussion

Given that high oral doses of metformin are required to achieve anticancer effects, the development of novel compounds derived from the active metformin structure has become an area of interest 16, 28. Our laboratory is committed to the development of novel biguanide derivatives with potent anticancer activities 15, 27. The results of previous work confirmed that the novel biguanide derivative **YB-004** has promising antiproliferative activity in BC. Therefore, further investigation into the antitumor mechanism of **YB-004** holds crucial clinical significance.

The G0/G1 phase arrest of the cell cycle caused by biguanides has become recognized as an anticancer mechanism. This study confirmed that **YB-004**, a new biguanide derivative, clearly induces G0/G1 phase arrest in BC cells. Liu *et al.* suggested that abnormalities in cell cycle progression constitute one of the critical mechanisms of tumorigenesis, and regulators of cell cycle could thus become reasonable targets for anticancer therapy 29. Suski *et al.* suggested that targeting a single component of the cell cycle might be an effective anticancer strategy and discussed the potential of inhibiting different proteins related to the cell cycle in cancer therapy: for example, the protein Cyclin D1 is highly expressed in a variety of cancers, including BC 30. Amplification of CCND1 has been observed in approximately 10% of all BC patients, and because of its potential as a prognostic biomarker and therapeutic target, CCND1 has garnered significant attention from researchers. However, the literature on CCND1 amplification and cyclin D1 expression is contradictory regarding their clinical significance and correlation with disease stage, indicating the need for more studies to determine the mechanisms by which they deeply regulate tumorigenesis and development 31, 32. Furthermore, while there are currently no approved therapies or drugs directly targeting CCND1 or cyclin D1 in UC, several clinical trials targeting CDK4/CDK6 in the cyclin D1 pathway are ongoing. Thus, treatments targeting cyclin D1 have the potential to be innovative breakthroughs in cancer treatment 31, 33, 34. Current challenges include identifying specific cyclin-dependent tumor subsets and delineating the full function of cyclins in cancer therapy.

The cell cycle is closely related to DNA damage repair, and finding the key molecules connecting these two factors has attracted extensive attention 35, 36. Cell cycle proteins have been shown to mediate the initiation and progression of DNA damage repair, and some of them are directly involved as substrates in DNA damage repair 37. Furthermore, the development of therapies utilizing the cell cycle mediated DNA damage response (DDR) is generally recognized as having important clinical value. Here, we discovered that Cyclin D1 is a novel regulatory protein for HR in BC cells. When DNA damage occurs, the nuclear accumulation of RAD51 promotes HR repair. The current consensus is that RAD51 is upregulated when genomic instability to ensure sufficient nuclear accumulation levels, which is a prerequisite for the initiation of HR 38. However, the specific mechanisms regulating RAD51 expression under genotoxic conditions remain unclear. In this study, we found that Cyclin D1 is a new post-translational modifier of RAD51, and the novel biguanide **YB-004** promotes the proteasomal degradation of RAD51 by inhibiting Cyclin D1. However, whether there are ubiquitin ligases or deubiquitinases directly involved in the **YB-004**-mediated regulation of RAD51 ubiquitination through Cyclin D1 requires further investigation. DDR therapy has emerged as a cancer-targeted treatment approach in recent years, with the successful use of PARP inhibitors to treat a subset of tumors with specific HR gene mutations on the basis of the theory of DNA damage repair 39, 40. The expansion of the population benefiting from the use of PARP inhibitors and overcoming the adaptive resistance caused by the long-term use of PARP inhibitors are popular issues in current research.

Given the development of DDR therapy based on the concept of “synthetic lethality,” PARP inhibitors are promising new options in the search for new small-molecule targeted therapies for BC. However, monotherapy with PARP inhibitor has no significant effect on advanced urothelial carcinoma regardless of HR status 41-43. We found that the intensity of DNA damage after 48 hours of olaparib treatment was significantly weaker than that after 24 hours through the comet assay, indicating that the DNA damage induced by olaparib in HR-proficient BC cells can be self-repaired by the cells. These findings suggest that some potential factors may regulate HR in UC, allowing cancer cells to survive by performing active HR in the presence of PARP inhibitors. Accordingly, inhibiting HR activity as much as possible is one way to expand the clinical application of PARP inhibitors to improve their “synthetic lethal” effects in different cancers. In this study, we identified Cyclin D1 as a key factor regulating HR. Thus, the immunofluorescence results for γ-H2AX indicated that silencing Cyclin D1 led to a decrease in Rad51 expression, impairing the effective repair of olaparib-induced DNA damage, resulting in increased DNA damage at 48 hours compared to 24 hours. HR occurs only during the S and G2/M phases. Therefore, targeting proteins that drive the cell cycle from the G1 phase to the S phase represents a strategic breakthrough in decreasing HR activity in BC cells. This study demonstrated that the Cyclin D1 protein was highly expressed in HR-proficient BC cells, and it may regulate the occurrence and development of BC by affecting the activity of HR. Interestingly, **YB-004** inhibited cyclin D1 expression, thereby interfering with the cell cycle of cancer cells and inhibiting HR, resulting in the HR-deficient phenotype of cancer cells. Our results confirmed that the YB-004 down-regulated the expression of Cyclin D1 and promoted the proteasomal degradation of Rad51, thereby blocking HR. Therefore, the combination of YB-004 and PARPi leads to the "synthetic lethality" of HR-proficient BC cells. These findings may provide insights into various types of cancers with high expression of Cyclin D1, including colon cancer and osteosarcoma.

In conclusion, the innovation of this study is following the concept of “synthetic lethality” to expand the clinical application of PARP inhibitors by combination with **YB-004**, a novel biguanide with anti-HR activity, providing a new way for BC treatment.

## Supplementary Material

Supplementary figures and tables.

## Abbreviations

BC: Bladder cancer
HR: Homologous recombination
CCND1: Cyclin D1
UC: Urothelial cancer
PARP: Poly (ADP-ribose) polymerase
H&E: hematoxylin and eosin
DDR: DNA damage reaction
