# Supplementary material for: Inhibition of Cyclin D1 by Novel Biguanide Derivative YB-004 Increases the Sensitivity of Bladder Cancer to Olaparib via Causing G0 / G1 Arrest
Source: Int J Biol Sci. 2025 Feb 18;21(5):1984–98. doi: 10.7150/ijbs.105072 (PMC11900825; doi:10.7150/ijbs.105072)
Supplement: Supplementary file 1 — Supplementary figures and tables. [file ijbsv21p1984s1.pdf]

Supplementary materials

Inhibition of cyclin D1 by novel biguanide derivative YB-004 increases the sensitivity of bladder cancer to Olaparib via causing G0 / G1 arrest

Di Xiao[1, 3#], Xuetong Chu[1#], Weifan Wang[1], Mei Peng[1], Qi Lv[1], Cangcang Xu[1]*, Huaxin Duan[1]*, Xiaoping Yang[1, 2]*

[1]Key Laboratory of Study and Discovery of Small Targeted Molecules of Hunan Province, Department of Oncology, Hunan Provincial People's Hospital, The First Affiliated Hospital of Hunan Normal University, The Research Center of Reproduction and Translational Medicine of Hunan Province, Key Laboratory of Chemical Biology & Traditional Chinese Medicine Research of Ministry of Education, Department of Pharmacy, School of Medicine, Hunan Normal University, Changsha 410013, Hunan, China; [2]FuRong Laboratory, Changsha 410078, Hunan, China; [3]TCM and Ethnomedicine Innovation and Development International Laboratory, Innovative Material Medical Research Institute, School of Pharmacy, Hunan University of Chinese Medicine, Changsha, China.

# These authors contributed equally to this work.

*Corresponding author: Cangcang Xu (xucangcang@hunnu.edu.cn), Huaxin Duan (huaxinduan_123@sina.com), Xiaoping Yang (xiaoping.yang@hunnu.edu.cn)

Contents

Supplementary Figures……………………………………………………………pages 3-6

Supplementary Tables……………………………………………………………pages 7-8

Figure S1

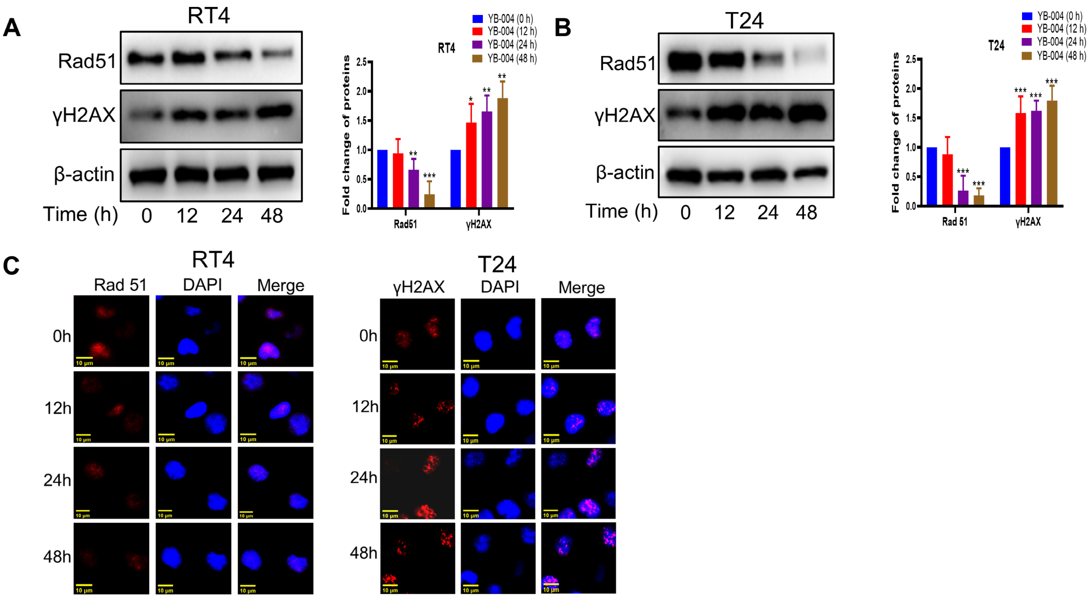

Figure S1. YB-004 inhibited HR of HR-proficient BC cells exacerbating DNA damage. A-C. Cells were treated with **YB-004**, and the changes of the indicated proteins were analyzed by WB and immunofluorescence (n=3, Error bars represent means ± SD from triplicate experiments, *P < 0.05; **P < 0.01; ***P < 0.001).

Figure S2

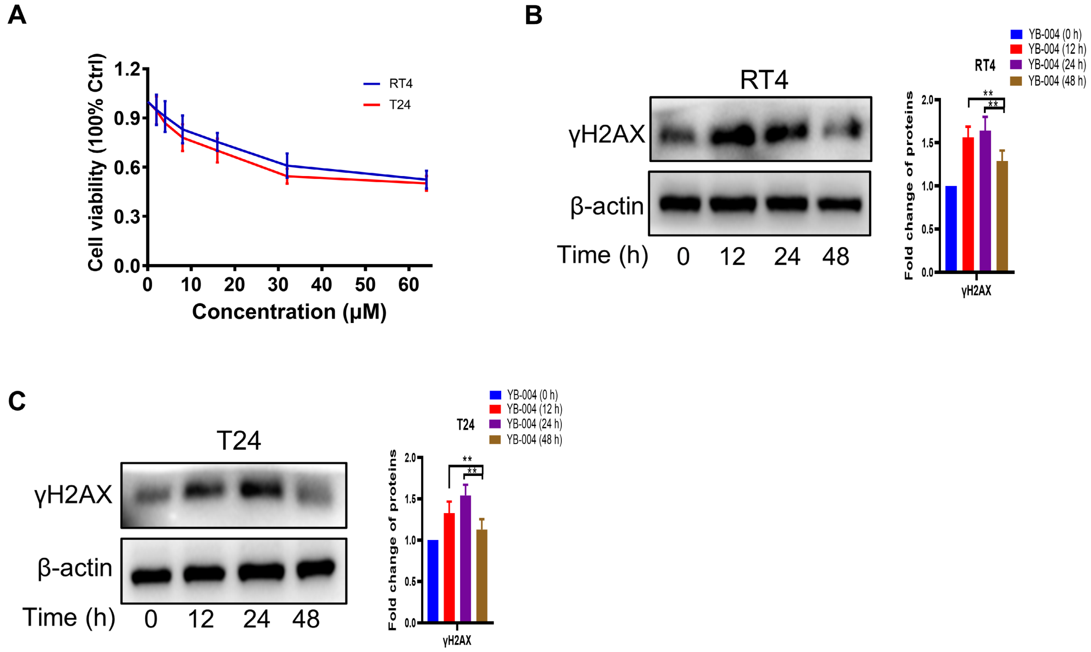

Figure S2. The effects of Olaparib on bladder cancer cells. A. The cell viability after Olaparib treatment were evaluated by MTT assay. **B-C**. RT4 (B) and T24 (C) were treated with Olaparib, and the changes of the indicated proteins were analyzed by WB (n=3, Error bars represent means ± SD from triplicate experiments, **$P < 0.01$).

Figure S3

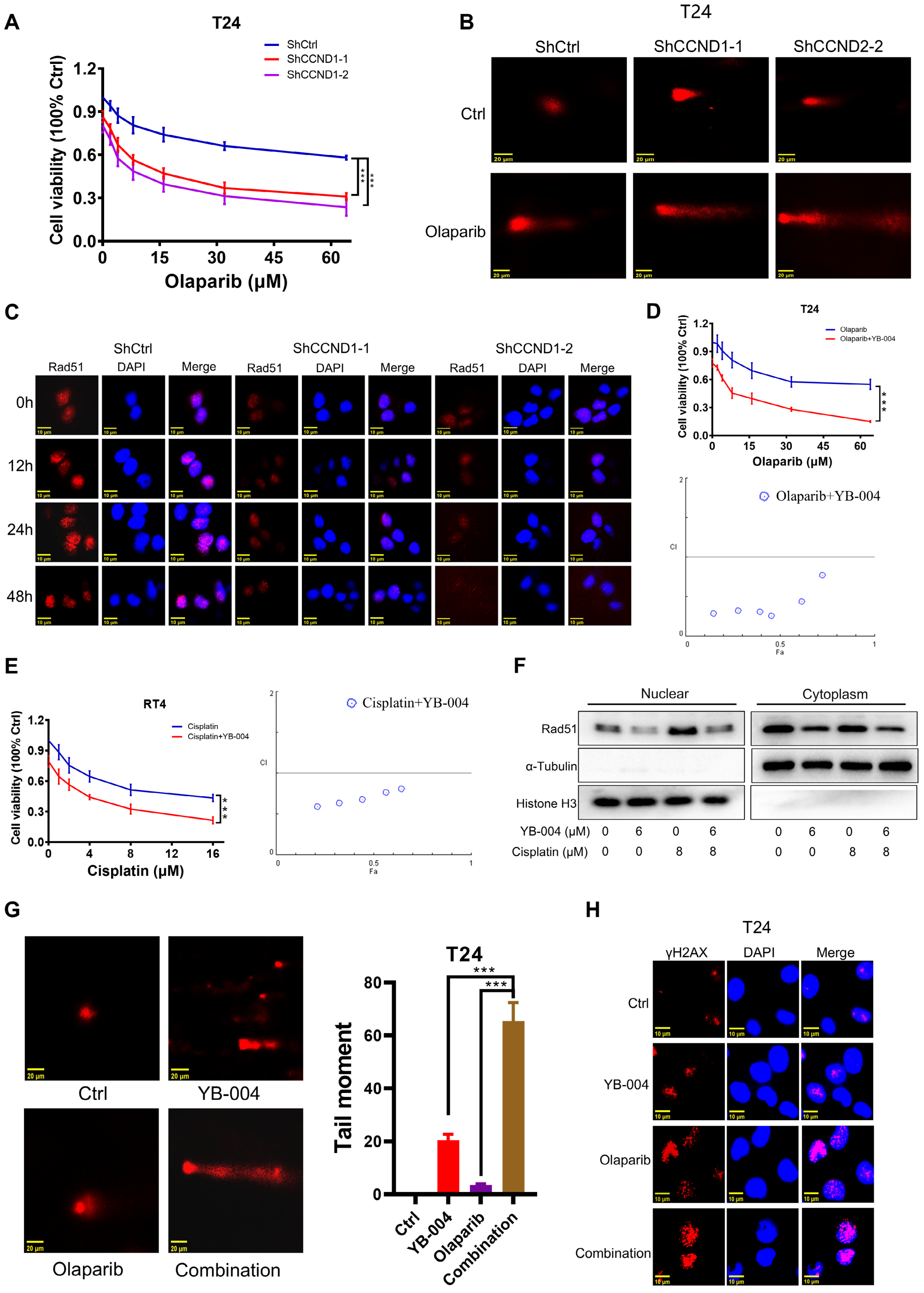

Figure S3. Combination of YB-004 and Olaparib synergistically inhibits the growth of HR-proficient BC cells in vitro. A. The cell viability of T24 expressing shCtrl or shCCND1 treated with Olaparib at different concentrations was evaluated by MTT. **B**. T24 expressing shCtrl or shCCND1 were treated with indicated concentrations of Olaparib for 24 h and the degree of DNA damage was measured by comet assay. **C**. BC cells expressing shCtrl or shCCND1 were

treated with Olaparib and the changes of the indicated proteins were analyzed by immunofluorescence. **D**. The cell viability of T24 treated with **YB-004** and Olaparib alone or in combination were evaluated by MTT. The combination index (CI) was calculated using CompuSyn software. **E**. The cell viability of RT4 treated with **YB-004** and cisplatin alone or in combination were evaluated by MTT. The combination index (CI) was calculated using CompuSyn software. **F.** RT4 were treated with **YB-004** or cisplatin and separated into nuclear and cytoplasmic fractions. These fractions were then detected by WB. **G**. T24 were treated with **YB-004** and Olaparib alone or in combination for 24 h and the degree of DNA damage was measured by comet assay. **H.** T24 were treated with **YB-004** and Olaparib alone or in combination and the changes of the indicated proteins were analyzed by immunofluorescence ($n=3$, Error bars represent means ± SD from triplicate experiments, ***$P < 0.001$).

Table S1. Primers for human genes in RT-PCR

Gene	Forward Primer(F)	Reverse Primer(R)
CCND1	GCGGAGGAGAACAAACAG	GCGGTAGTAGGACAGGAA
CCND2	CCGACAACTCCATCAAGCCTCAG	TGCCAGGTTCCACTTCAACTTCC
CCND3	AGCCTCAGGGAAGCCTCTCAG	CATCTGTAGGAGTGCTGGTCTGG
CCNE	TGTCCTGGATGTTGACTGCCTTG	TTCTCTATGTCGCACCACTGATACC

Table S2. shRNA sequences for the targeted genes

Gene	Forward Primer(F)	Reverse Primer(R)
Sh-CCND1-1	CcggGAACAAACAGATCATCCGCAACTCGAGTTGCGGATGATCTGTTTGTTCTTTTTg	aattcaaaaaGAACAAACAGATCATCCGCAACTCGAGTTGCGGATGATCTGTTTGTTC
Sh-CCND1-2	CcggGCACGATTTCATTGAACACTTCTCGAGAAGTGTTCAATGAAATCGTGCTTTTTg	aattcaaaaaGCACGATTTCATTGAACACTTCTCGAGAAGTGTTCAATGAAATCGTGC
Sh-NC	CCGGTTCTCCGAACGTGTCACGTTTCAAGAGAACGTGACACGTTCGGAGAATTTTTG	AATTCAAAAATTCTCCGAACGTGTCACGTTCTCTTGAAACGTGACACGTTCGGAGAA
